# The correlation of sperm morphology with unexplained recurrent spontaneous abortion: A systematic review and meta-analysis

**DOI:** 10.18632/oncotarget.17233

**Published:** 2017-04-19

**Authors:** Xiaodan Cao, Yun Cui, Xiaoxia Zhang, Jiangtao Lou, Jun Zhou, Renxiong Wei

**Affiliations:** ^1^ Department of Clinical Laboratory, Ningbo Municipal Hospital of Traditional Chinese Medicine, Ningbo 315000, China

**Keywords:** sperm, morphology, recurrent spontaneous abortion, meta-analysis

## Abstract

Sperm morphology displays a potential impact on sperm function and may ultimately impact reproductive function. Current studies have investigated the correlation between sperm morphology with unexplained recurrent spontaneous abortion (RSA) but have shown inconsistent results. Hence, we systematically searched MEDLINE, EMBASE, CNKI databases, as well as the Cochrane Library for studies that examined the association between sperm morphology and unexplained RSA. Fifteen studies were identified, including 883 cases and 530 controls. Our meta-analysis results indicated that the percentage of normal sperm morphology from men with RSA partners was significantly lower than those from normal controls(SMD [95% CI]: − 0.60 [−0.81, −0.40]; P<0.00001) and the percentage of sperm morphologic alterations was significantly higher in patients with RSA compared with the control group (SMD [95% CI]: 0.92 [0.42, 1.43]; P=0.0004). The present study suggested that the percentage of normal sperm morphology may indeed decrease in men from RSA group compared with controls. However, there were some limitations in the study such as the differences in stain techniques and classification criteria. Further evidences are needed to better elucidate the relationship between sperm morphology and unexplained RSA.

## INTRODUCTION

Recurrent spontaneous abortion (RSA), defined as a couple having two or more consecutive pregnancy losses in the first or early second trimester of gestation [1], affects approximately 0.8%–1.4% of couples trying to conceive and is one of the most frustrating and difficult areas in reproductive medicine [2]. Due to complex causes involved in pregnancy loss and few evidence-based diagnostic strategies, the etiology of RSA remains unexplained in more than half of those affected [3]. Unexplained RSA was diagnosed after exclusion of the causes such as infections, immunologic problems, genetic anomalies, hormonal disorders and abnormal anatomic structures. RSA is usually approached from maternal factors owing to the intimate maternal relationship with the developing embryo. Recently, more attention has been paid to the effect of male factors on RSA [4, 5].

Clinical laboratory examination of men with RSA partners involves a routine semen analysis to assess the semen parameters such as sperm concentration, motility, viability and morphology. Sperm morphology provides important information regarding the process of spermiogenesis that displays a potential impact on sperm function and may ultimately impact reproductive function [6]. In the late 1980’s, Dr. Kruger first proposed the idea that sperm morphology contributed to reproductive success and illustrated that abnormal sperm morphologies were associated with fertilization failure in couples attending an *in vitro* reproductive performance [7]. In the late 1990’s, Bonde et al. reported that men with abnormal sperm morphologies had a decreased possibility of achieving pregnancy [8]. The percentage of normal sperm morphology was important predictor of probability of conception and there existed a direct correlation between normal sperm morphology and time to pregnancy [9, 10]. The morphology of sperm head has a major impact on sperm hydrodynamic efficiency [11]. Only morphologically normal sperm were thought to have the ability to pass through the female reproductive tract and the zona pellucida of the egg [12–14]. Abnormal sperm morphology is also thought to be related with the increased levels of chromosomal abnormalities, sperm aneuploidy and DNA fragmentation, and sperm morphology has been described as one of the major determinants of male *in vivo* and *in vitro* fertility [15–17]. Therefore, the assessment of sperm morphology is an important part of male fertility assessment, which provides valuable insight into the quality of semen and exhibit comprehensive information of the reproductive function.

Sperm morphology has been questioned by some researchers about its real value as a prognostic factor for unexplained RSA. Attempts to show a correlation between couples with RSA and the fertile men in terms of sperm morphology have been debatable. Despite most studies in this area, there is no consensus regarding the effect of abnormal sperm morphology on the risk of recurrent abortion. One group suggested that men from the control group had a significantly higher percentage of normal sperm morphology compared with men from the RSA group. In contrast, other studies reported that there was no significant difference in the percentage of normal sperm morphology between controls and men with RSA partners. Based on this controversy, the objective of our study was to investigate the relationship between sperm morphology and unexplained RSA by performing a systematic review and meta-analysis.

## RESULTS

### Characteristics of the included studies

**Figure 1:**
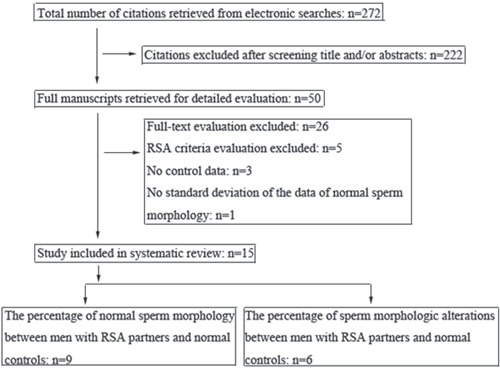
Flow chart showing the selection of eligible studies

The search strategy yielded 272 citations according to the eligibility criteria. Of these, 222 publications were excluded after screening title or abstracts due to the irrelevant contents and 50 studies were retrieved for further evaluation. Of the 50 remaining publications, 26 were excluded after full-text evaluation. Five studies were excluded as they did not fulfil the RSA criteria. Three studies were excluded as they had no control data and one study was excluded as no standard deviation of the data of normal sperm morphology was reported. Therefore, the total number of studies included in the review was 15 and 883 couples with recurrent abortion and 530 fertile couples as normal controls were included (Figure 1). Table 1 summarized the main characteristics of the 15 citations and the types of these citations were case control studies. The studies scored well on the Newcastle–Ottawa Quality Assessment Scale (NOS score > 5) and the assessment result of sperm morphology was expressed as a percentage of normal morphology or morphologic alterations.

**Table 1:** Characteristics of the included studies investigating the relationship between sperm morphology and unexplained recurrent spontaneous abortion

Study	Country	Number (cases/controls)	Mean age (cases/controls)	Assessment result	Classification criteria	Stain technique	NOS
Jiang 2011	China	62/40	NI	normal morphology	WHO 2001	Papanicolaou stain	8
Liu 2010	China	56/56	NI	normal morphology	WHO	Papanicolaou stain	6
Ma 2015	China	62/35	(33.6±4.1)/(32.2±3.9)	normal morphology	WHO	Diff-Quik	7
Wang 2013	China	68/63	NI	normal morphology	WHO 1999	Papanicolaou stain	7
Zhang 2012a	China	111/30	NI	normal morphology	WHO 1999	Diff-Quik	8
Zhang 2012b	China	40/40	(28.9±3.7)/(29.4±4.5)	normal morphology	WHO 1999	NI	8
Gil-Villa 2010	Colombia	23/11.	(37.9±6.5)/(30.0±6.6)	normal morphology	Kruger strict criteria	NI	7
Nabi 2013	Iran	30/30	(31.97±4.45)/(31.43±7.00)	normal morphology	WHO 2010	Papanicolaou staining	8
Talebi 2016	Iran	40/40	(35±6)/(35±6)	normal morphology	WHO 1999	Papanicolaou staining	8
Gong 2015	China	84/62	NI	morphologic alterations	WHO	NI	6
Hill 1994	Massachusetts	98/17	NI	morphologic alterations	WHO 1992	eosin and thiazine	8
Kazerooni 2009	Iran	30/30	(34.6±5.6)/(33.8±6.3)	morphologic alterations	Kruger strict criteria	hematoxylin staining	8
Sbracia1 1996	Italy	120/30	(36.7±4.9)/(35.8±3.1)	morphologic alterations	WHO 1987	eosin and thiazine	7
Zhang 2009	China	37/26	NI	morphologic alterations	Kruger strict criteria	Papanicolaou stain	8
Zidi-Jrah 2016	Tunisia	22/20	(37.1±5.4)/(36.9±5.73)	morphologic alterations	WHO 2010	NI	7

The assessment result of sperm morphology was expressed as a percentage of normal morphology or morphologic alterations. NI: not indicated in the study.

### Meta-analysis

**Figure 2:**
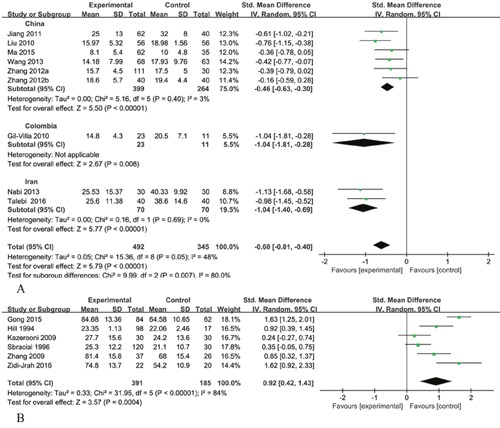
Meta analysis **(A)** Forest plot showing the meta-analysis outcomes of the percentage of normal sperm morphology between men with RSA partners and normal controls. **(B)** Forest plot showing the meta-analysis outcomes of the percentage of sperm morphologic alterations between men with RSA partners and normal controls. IV: inverse variance; Random: random-effects model.

Nine studies reported the percentage of normal sperm morphology. Pooling the results of the nine studies showed that the percentage of normal sperm morphology was significantly lower in patients with RSA compared with normal controls (SMD [95% CI]: − 0.60 [− 0.81, − 0.40]; P<0.00001). Statistical heterogeneity was found between the studies (I^2^ = 48%; P = 0.05) and a random effects model was applied for pooling of the results. In the subgroup meta-regression analysis, the SMD [95% CI] in China subgroup was − 0.46 [− 0.63, − 0.30] and the SMD [95% CI] in Iran subgroup was −1.04 [− 1.40, − 0.69]. The percentage of normal sperm morphology was significantly lower in patients with RSA compared with controls (P<0.00001) and there was no statistical heterogeneity in the both subgroups (I^2^ = 3%, P = 0.4; I^2^ = 0%, P = 0.69) (Figure 2A).

Six studies reported the percentage of sperm morphologic alterations. Statistical heterogeneity was observed between the studies (I^2^ = 84%; P<0.00001) and a random effects model was used. Pooling the results of the six studies showed the percentage of sperm morphologic alterations was significantly higher in patients with RSA compared with the control group (SMD [95% CI]: 0.92 [0.42, 1.43]; P=0.0004) (Figure 2B).

### Publication bias

**Figure 3:**
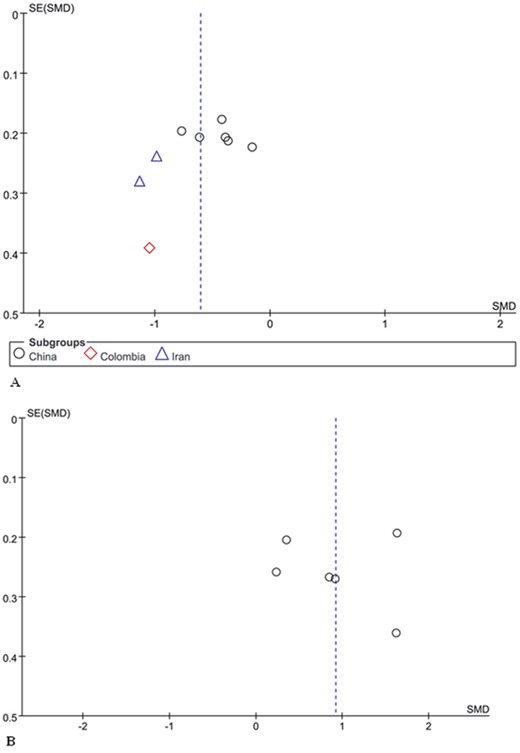
Funnel plot analysis **(A)** Funnel plot of the percentage of normal sperm morphology between men with RSA partners and normal controls. **(B)** Funnel plot of the percentage of sperm morphologic alterations between men with RSA partners and normal controls.

**Figure 4:**
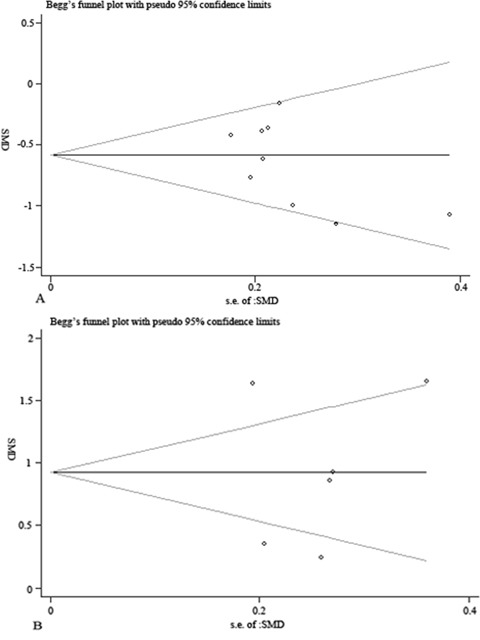
Begg's publication bias analysis **(A)** Begg's publication bias plot of the percentage of normal sperm morphology between men with RSA partners and normal controls. **(B)** Begg's publication bias plot of the percentage of sperm morphologic alterations between men with RSA partners and normal controls. The funnel plot did not show any substantial asymmetry, suggesting no evidence of publication bias.

The funnel plot showed no evidence of publication bias of the meta-analysis owing to its symmetrical shape (Figure 3), although a small study might have been missed. Begg's and Egger's test of publication bias of the sperm morphology in males with RSA partners and control males indicated a lack of publication bias (*P*>0.05) (Figure 4, Table 2).

Table 2Egger’s test of publication bias

**Table:** A

Std_Eff	Coef.	Std. Err.	t	P > |t|	[95% Conf. Interval]
slope	0.26	0.49	0.52	0.62	−0.90 1.41
bias	−3.87	2.20	−1.75	0.12	−9.09 1.35

**Table:** B

Std_Eff	Coef.	Std. Err.	t	P > |t|	[95% Conf. Interval]
slope	0.88	1.46	0.61	0.58	−3.17 4.94
bias	0.16	5.98	0.03	0.98	−16.46 16.78

A. Egger's test of the percentage of normal sperm morphology between men with RSA partners and normal controls.

B. Egger's test of the percentage of sperm morphologic alterations between men with RSA partners and normal controls.

### Sensitivity analysis

**Figure 5:**
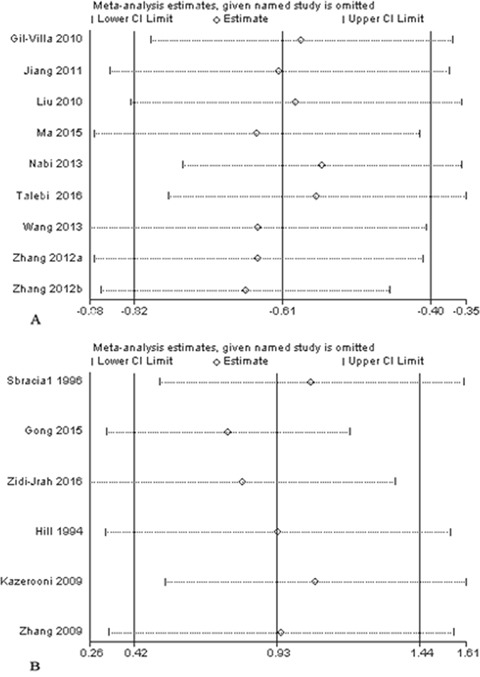
Sensitivity analysis **(A)** Sensitivity analysis plot of the percentage of normal sperm morphology between men with RSA partners and normal controls. **(B)** Sensitivity analysis plot of the percentage of sperm morphologic alterations between men with RSA partners and normal controls.

The calculated combined SMD remained consistent when we omitted each study sequentially. In the meta-analysis, none of an individual study significantly changed the combined SMD results, suggesting the results were statistically stable (Figure 5).

## DISCUSSION

In our study, fifteen articles studied the association between sperm morphology and unexplained RSA. Nine studies reported the assessment results of sperm morphology as the percentage of normal morphology, of which five studies indicated that the percentage of normal sperm morphology from RSA group were significantly lower than those from control group and four study showed no significant difference between RSA group and controls. We subanalysed the data with regard to the country of patients included and found that the subgroups showed a significant decrease in the percentage of normal morphology with men from RSA group with no statistical heterogeneity. Six studies reported the assessment results of sperm morphology as the percentage of morphologic alterations, of which four studies indicated that men from RSA group had a significantly higher percentage of morphologic alterations compared with controls and two study showed no significant difference in the percentage between controls and men with RSA partners. Our meta-analysis has demonstrated that abnormal sperm morphology is significantly correlated with the unexplained RSA.

The potential mechanism of the effects of abnormal sperm morphology on recurrent abortion is a complicated problem. Morphologically abnormal spermatozoas and semina leucocytes are the main sources of reactive oxygen species (ROS) [18]. The increased levels of ROS may damage other sperm structure and lead to additional aspects of sperm dysfunction such as reduced motility or high levels of DNA damage during spermatogenesis, thus impairing their capacity to fertilize [19, 20]. Several studies demonstrated that a high percentage of abnormal sperm morphology was correlated with embryo failure at an early cleavage stage and it was thought that defective DNA in sperm influenced the adequate expression and regulation of paternal genes in early embryos [21–23]. Sperm quality may adversely affect early embryonic development by some possible ways, including abnormal DNA and abnormal sperm membrane protein [24–27]. Seli et al. reported that nuclear DNA integrity in sperm was associated with embryo development at the blastocyst stage, and the paternal genes played important roles in embryo function [28]. Ahmadi et al. reported that sperm with abnormal DNA could accomplish fertilization of oocyte and generate high-quality early-stage embryos in a mouse model, however, as the extent of the DNA fragmentation increases, the possibility of successful pregnancy decreases [29]. It is widely supposed that embryo development is subjected to maternal control at the early steps and that the paternal genes affect the development at the 4- to 8-cell stage [5]. Therefore, at this stage, the consequences of paternal DNA damage may become apparent, impairing embryonic development [30]. One theory of a possible cause of RSA is a defect in the inhibition of implantation of “poor quality” embryos [31]. Sperm morphology may affect the quality of spermatogenesis and other facets of sperm function [32], and there exist a possibility of morphologic alterations in sperm inducing RSA through causing sperm DNA damage and interfering the early embryo development.

In the clinical andrology laboratory, the causes of an identified sperm morphology defect were almost never defined. A conclusive relation between some factors and sperm morphology has not been demonstrated. With regard to male age, some studies have shown that with increasing paternal age, the percentage of normal sperm morphology tend to decrease [33–38]. Zhu et al. performed the semen analysis of 20–60 years old men and showed that age was negatively correlated with percentage of normal sperm morphology and it began to decline gradually at the age of 30 [37]. Kidd et al. found morphologically normal sperm rate declined at age of 40 and there existed a significant difference in morphologically normal sperm rate between two age groups (30y vs. 50y) [38]. Different studies on the relation between sperm morphology and common lifestyle have shown varying results. Some studies indicated that there were no significant correlation between sperm morphology with body mass index (BMI), obesity, smoking, alcohol consumption, type of underwear or having a history of mumps, suggesting the little impact of an individual's lifestyle on sperm morphology [39–42]. In contrast, Jeng et al. in Taiwan found that those smoking 10 cigarettes/day were less likely to have morphologically normal sperm and a study from New Zealand showed the association of better sperm morphology with increasing BMI [43, 44]. Genetic factors are suspected to play significant roles in the majority of morphologic alterations. Zona pellucida binding protein 1 (ZPBP1), which localizes to the acrosomal membrane and likely binds to the oocyte zona pellucida (ZP) protein, was related with sperm head morphology, and the association of mutations in ZPBP1 gene with abnormal sperm head morphology was demonstrated in a study by Yatsenko [45]. Oxidative stress is another factor that may cause disruption of spermatogenesis in the testes and the excessive production of reactive oxygen species (ROS) could contribute to decline observed in morphologically normal sperm [46, 47].

Antioxidants (such as vitamins and dietary supplements) are scavengers of ROS and therefore they have been suggested as a treatment to neutralize or reduce the ROS content and reverse the adverse effect of high levels of ROS on semen parameters [48, 49]. Several studies have showed a significant increase in sperm motility and spontaneous or assisted pregnancy rates with antioxidant use [48–52]. Men in RSA couples who presented an augmented production of free radicals or increased sperm DNA damage, could successfully accomplish their pregnancies during the first three months when antioxidants were used [53]. However, the existing data is still debatable and large-scale studies are required focusing on the impact of antioxidants on sperm parameters and their relationship with early embryo development.

Some limitations in our study need to be addressed. The review included studies that differed in characteristics, stain techniques and classification criteria. Important confounding factors, such as male age, were not always noted. The stain techniques for sperm morphology assessment were not detailed in some studies and varied in the rest. The different criterias (Kruger strict criteria versus WHO criteria) were employed to classify the normal/abnormal spermatozoa. Besides these factors, the subjectivity of morphology assessment might be another limiting factor and the classification of sperm morphology depended on the technician's concept of the definition of normality and the staining procedures [54–57]. The multiple steps required in the process could induce artifacts that might potentially contribute to the alteration of final interpretation [58].

In summary, the current evidences suggest that the percentage of normal sperm morphology may indeed decrease in men with RSA partners compared with normal controls. This information could make recommendations for reproduction diagnosis and treatment and could affect public health. However, this evidence is far from conclusive because of the small sample sizes of the currently available studies and because of the paucity of studies in treatment strategies. Further evidence gathered through well-designed and well-conducted trials to better elucidate the relation between sperm morphology and unexplained RSA is warranted.

## MATERIALS AND METHODS

### Literature search

Two independent reviewers searched the MEDLINE, EMBASE, CNKI databases, as well as the Cochrane Library, from inception until December 2016. The following search terms combined Medical Subject and Emtree headings and textwords were used to generate two subsets of citations, including terms on spontaneous abortion (pregnancy loss, miscarriage, spontaneous abortion, recurrent abortion, habitual abortion, embryo loss) and terms on sperm morphology (sperm, spermatozoa, morphology, sperm form). The two subsets were combined with ‘AND’ to generate a subset of citations relevant to our research question. The language or study type was not restricted.

### Criteria for study inclusion and exclusion

Unexplained RSA in this study was defined as a couple having two or more consecutive pregnancy losses in the first or early second trimester of gestation and absence of the clinical conditions: abnormal anatomic structures, hormonal disorders, infections, anti-nucleus antibodies, anti-phospholipid antibodies, anti-thyroid antibodies, hypofibrinogenaemia or thrombocytosis in the women, karyotyping abnormalities in both partners. Healthy men whose partners had achieved full-term pregnancies without any history of infertility or recurrent miscarriage were recruited as controls. Couples were not eligible for this study if male partners had a recent fever or exposure to pesticides, radiotherapy, chemotherapy, or heavy metals. None of the male partners had a history of alcohol consumption or drug abuse.

### Study selection and data extraction

Two independent reviewers scrutinized the titles and abstracts of articles from the electronic searches. All of the relevant studies that were likely to meet the predefined criteria were retrieved. Final inclusion or exclusion decisions were made by reviewing the full manuscripts. Disagreements were resolved by consensus or a third reviewer.

Two independent reviewers completed the quality assessment of observational studies using the Newcastle–Ottawa Quality Assessment Scales. Items assessed included selection of cases and controls, comparability of cases and controls, ascertainment of exposure. We used an arbitrary score based on the assumption of equal weight of all items included in the Newcastle–Ottawa Scale. This was used to give a quantitative quality evaluation of each study with a score ranged from 0 to 9. Data were extracted from the included studies and showed in Table 1.

### Statistical analysis

We pooled the standard mean differences (SMDs) of the percentage of normal sperm morphology or morphologic alterations from the individual studies with 95% confidence intervals. Heterogeneity was assessed graphically using Forest plots [59] and evaluated statistically by the P-value and I^2^ statistic to quantify the percentage of total variation across studies. If the P-value was less than 0.1, or the I^2^-value was greater than 50%, the summary estimate was analyzed in a random-effects model. Otherwise, a fixed-effects model was used [60]. Publication bias was assessed visually by the symmetry of funnel plots [61] and also detected using Begg's test and Egger's test. A sensitivity analysis was conducted to estimate the stability of the meta-analysis. Statistical analyses were performed using RevMan 5.3 software (Cochrane Collaboration, Copenhagen, Denmark) and Stata 11.0 software (College Station, Texas, USA).
